# Structure-Function Analysis of the Curli Accessory Protein CsgE Defines Surfaces Essential for Coordinating Amyloid Fiber Formation

**DOI:** 10.1128/mBio.01349-18

**Published:** 2018-07-17

**Authors:** Roger D. Klein, Qin Shu, Zachary T. Cusumano, Kanna Nagamatsu, Nathaniel C. Gualberto, Aaron J. L. Lynch, Chao Wu, Wenjie Wang, Neha Jain, Jerome S. Pinkner, Gaya K. Amarasinghe, Scott J. Hultgren, Carl Frieden, Matthew R. Chapman

**Affiliations:** a Department of Molecular Microbiology, Washington University School of Medicine, St. Louis, Missouri, USA; b Center for Women’s Infectious Disease Research (CWIDR), Washington University, School of Medicine, St. Louis, Missouri, USA; c Department of Biochemistry and Molecular Biophysics, Washington University School of Medicine, St. Louis, Missouri, USA; d Department of Molecular, Cellular, and Developmental Biology, University of Michigan, Ann Arbor, Michigan, USA; e Department of Pathology and Immunology, Washington University School of Medicine, St. Louis, Missouri, USA; The Ohio State University School of Medicine

**Keywords:** *Escherichia coli*, functional amyloid, nucleation-precipitation, bioassembly, biofilms, curli, extracellular matrix, nuclear magnetic resonance

## Abstract

Curli amyloid fibers are produced as part of the extracellular biofilm matrix and are composed primarily of the major structural subunit CsgA. The CsgE chaperone facilitates the secretion of CsgA through CsgG by forming a cap at the base of the nonameric CsgG outer membrane pore. We elucidated a series of finely tuned nonpolar and charge-charge interactions that facilitate the oligomerization of CsgE and its ability to transport unfolded CsgA to CsgG for translocation. CsgE oligomerization *in vitro* is temperature dependent and is disrupted by mutations in the W48 and F79 residues. Using nuclear magnetic resonance (NMR), we identified two regions of CsgE involved in the CsgE-CsgA interaction: a head comprising a positively charged patch centered around R47 and a stem comprising a negatively charged patch containing E31 and E85. Negatively charged residues in the intrinsically disordered N- and C-terminal “tails” were not implicated in this interaction. Head and stem residues were mutated and interrogated using *in vivo* measurements of curli production and *in vitro* amyloid polymerization assays. The R47 head residue of CsgE is required for stabilization of CsgA- and CsgE-mediated curli fiber formation. Mutation of the E31 and E85 stem residues to positively charged side chains decreased CsgE-mediated curli fiber formation but increased CsgE-mediated stabilization of CsgA. No single-amino-acid substitutions in the head, stem, or tail regions affected the ability of CsgE to cap the CsgG pore as determined by a bile salt sensitivity assay. These mechanistic insights into the directed assembly of functional amyloids in extracellular biofilms elucidate possible targets for biofilm-associated bacterial infections.

## IMPORTANCE

Curli represent a class of functional amyloid fibers produced by Escherichia coli and other Gram-negative bacteria that serve as protein scaffolds in the extracellular biofilm matrix. Despite the lack of sequence conservation among different amyloidogenic proteins, the structural and biophysical properties of functional amyloids such as curli closely resemble those of amyloids associated with several common neurodegenerative diseases. These parallels are underscored by the observation that certain proteins and chemicals can prevent amyloid formation by the major curli subunit CsgA and by alpha-synuclein, the amyloid-forming protein found in Lewy bodies during Parkinson’s disease. CsgA subunits are targeted to the CsgG outer membrane pore by CsgE prior to secretion and assembly into fibers. Here, we use biophysical, biochemical, and genetic approaches to elucidate a mechanistic understanding of CsgE function in curli biogenesis.

## INTRODUCTION

Bacterial biofilms are a major virulence factor in many infections, including indwelling venous catheter sepsis, prosthetic-valve infective endocarditis, and Foley catheter-associated urinary tract infections (CAUTIs) (1–3). Biofilm bacteria assemble an extracellular matrix that helps protect bacteria from environmental stressors, including desiccation, oxidative damage, and shear stress (4, 5). Common components of the biofilm extracellular matrix are amyloids, a class of proteins that form SDS-insoluble fibrils 4 to 7 nm thick with a characteristic cross β-strand architecture that provides structural integrity to the fibrils (6, 7). In contrast to pathogenic amyloids, such as amyloid beta (Aβ) in Alzheimer’s disease and α-synuclein in Parkinson’s disease, these functional amyloids are produced as the result of highly coordinated biosynthetic processes (8). Curli are among the best-studied functional amyloids and are an integral part of the biofilm extracellular matrix produced by *Salmonella*, *Enterobacteriaceae*, and Escherichia coli (9). Solid-state nuclear magnetic resonance (NMR) studies have found that curli account for 85% of the total biofilm extracellular matrix, with polysaccharides constituting a majority of the remaining 15% (10). In addition to their structural role in maintaining the biofilm architecture, curli fibers regulate inflammatory and immune responses in the gut, in part by decreasing the permeability of tight junctions in the intestinal epithelium (11, 12). Additionally, curli fibers can be engineered to scaffold a variety of functional biomolecules, with applications ranging from the delivery of self-sustaining therapeutics to environmental bioremediation (13–15).

**FIG 1:**
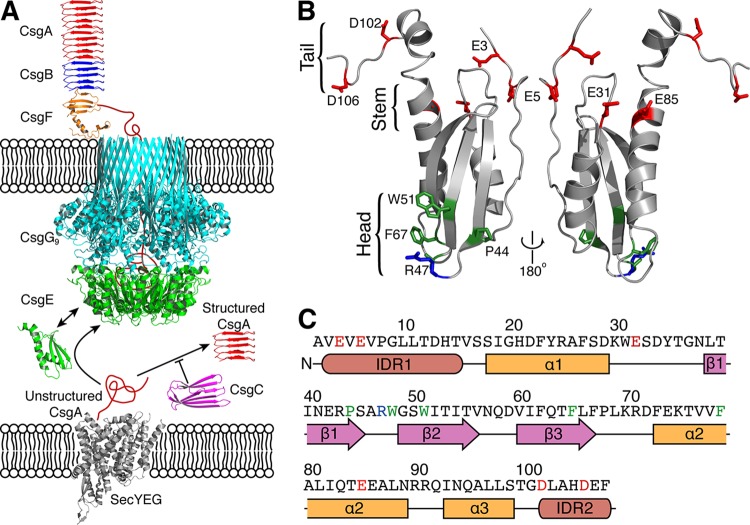
Working model for curli biogenesis. (A) Unstructured CsgA transported into the periplasmic space by the Sec translocon is maintained in a soluble, secretion-competent state by CsgC prior to delivery to the CsgG pore by the periplasmic accessory protein CsgE. Once secreted to the outer membrane, CsgA adopts an amyloid fold and is assembled into fibers by the action of CsgF and CsgB. (B) A positively charged cluster of residues (residues R43 and R47 and residues K70 and R71) define the “head” of the molecule, while negatively charged residues (E3, E5, D102, and D106) are enriched at the disordered “tail” of the molecule. (C) Primary sequence of CsgE, with positive, negative, and hydrophobic residues highlighted in blue, red, and green, respectively.

The proteins involved in the structure and biogenesis of curli fibers are encoded by two divergently transcribed operons: *csgBAC* and *csgDEFG* (16). Structurally, curli fibers are primarily composed of CsgA, a 13-kDa, β-rich protein (8, 17). These subunits are assembled into fibers *in vivo* by a mechanism known as nucleation-precipitation, sometimes referred to as the type VIII secretion system (outlined in Fig. 1A) (18). Briefly, CsgA, following translation, is translocated to the periplasm via the SecYEG complex, where it is protected from premature polymerization by the chaperone-like CsgC protein (19). CsgC maintains CsgA in an amorphous, β-strand-deficient state to prevent toxicity to the host cell by inhibiting primary nucleation and/or elongation through a series of electrostatic interactions (20–22). Periplasmic CsgA is then targeted to an outer membrane pore composed of nine identical CsgG subunits, each of which contributes four β-strands to a 36-strand transmembrane β-barrel and three residues to a series of rings that constrict the secretion channel diameter to 9 Å, a size consistent with the expulsion of unfolded, soluble CsgA into the extracellular space (23–25). Once secreted, CsgA fiber formation and elongation are nucleated by CsgB on the cell surface in a CsgF-dependent manner (26–29). Although no periplasmic CsgA is detected upon deletion of *csgG*, concomitant deletion of *csgG* and *csgC* results in the accumulation of CsgA aggregates in the periplasm (17, 19). These findings, coupled with the absence of intrinsic CsgC proteolytic activity, suggest that CsgC helps to maintain CsgA in a protease-sensitive state (19).

The accessory periplasmic protein CsgE is necessary for the transport of curli structural components through the CsgG pore (30). CsgE is thought to bind unfolded CsgA in the periplasm and deliver it to CsgG, where CsgE assembles into a nonameric ring that “caps” the periplasmic vestibule of the outer membrane pore (23, 31). Identification of CsgE’s putative role as a gating and specificity factor for the CsgG pore is based on direct biochemical and biological evidence. Coimmunoprecipitation studies have demonstrated a stable physical interaction between CsgE and CsgG (24). In addition, a complex consisting of 9 subunits each of CsgE and CsgG has been observed using cryo-electron microscopy (cryo-EM) (23). Single-channel current recordings and *in vivo* toxicity assays reveal that the presence of CsgE decreases the permeability of the CsgG channel with respect to ions and small molecules in a manner dependent on the CsgE concentration (23). CsgE can also differentially regulate protein translocation through the CsgG channel (30). Translocation of nonnative client proteins is attenuated in the presence of CsgE, while translocation of curli proteins containing the 20-to-21-residue N-terminal CsgG localization sequence is unaffected (30). Direct biochemical evidence for a CsgE-CsgA interaction has not been previously reported, although CsgE can delay CsgA amyloid formation *in vitro*, suggesting an interaction between CsgE and CsgA (30).

CsgE contains intrinsically disordered regions (IDRs) and readily forms oligomers and high-molecular-weight aggregates, making the atomic-resolution structure of wild-type (WT) CsgE challenging to obtain (32). However, the structure of the CsgE W48A/F79A mutant has been determined (Biological Magnetic Resonance Data Bank accession number 25927) (Fig. 1B and C) (33). CsgE W48A/F79A is functional in curli biogenesis *in vivo* and behaves similarly to WT CsgE in *in vitro* amyloid polymerization assays. Thus, the structure of CsgE W48A/F79A provides insight into surface-exposed residues that mediate interactions with CsgA, CsgG, and itself during homo-oligomerization (33). Examination of the electrostatic surfaces on CsgE revealed three regions of concentrated charge: (i) a positively charged “head,” including R47, K70, and R71; (ii) a negatively charged “stem,” including E31, D33, and E85; and (iii) a negatively charged “tail,” including E3, E5, D102, D106, and E107 in the N- and C-terminal IDRs (Fig. 1B). When the CsgE NMR structure was fitted into a low-resolution cryo-EM density map, two possible CsgE orientations were observed: one in which the disordered tails coalesced in the center of the disc and one in which the positively charged heads formed the center (33).

In this study, we utilized solution-state NMR and mutagenesis approaches to elucidate the molecular basis of the CsgE-CsgA, CsgE-CsgG, and CsgE-CsgE interactions. We found that the R47 residue at the head of CsgE mediates an indispensable charge-charge interaction with CsgA that is abrogated upon mutation. Conversely, mutations at the stem of CsgE augment the CsgE-CsgA interaction but decrease the efficiency of curli assembly *in vivo*. We also found that the CsgE-CsgA interaction is impervious to disruption by mutations in the head, stem, or tail residues of CsgE. Finally, we observed a temperature dependence of CsgE oligomerization that can be altered by mutations to residues W48 and F79.

## RESULTS

### NMR chemical shift perturbations (CSP) reveal key residues in the CsgE-CsgA interaction.

**FIG 2:**
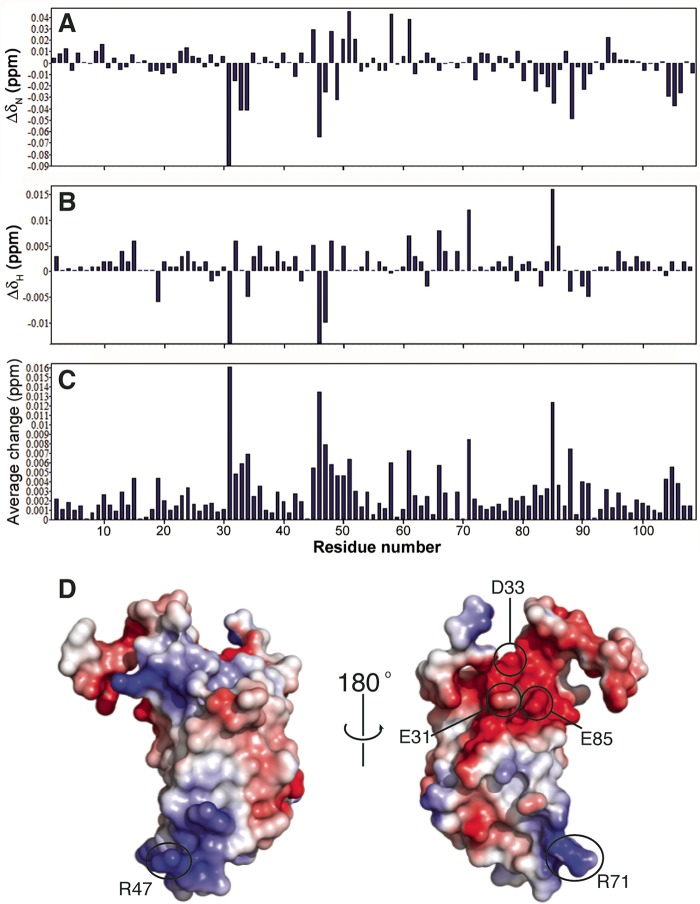
Chemical shift perturbations of ^15^N-labeled CsgE W48A/F79A by CsgA. (A to C) Chemical shift changes of (A) ^15^N and (B) ^1^H and (C) the average change of Euclidean distance (in parts-per-million units). (B) Structural mapping of chemical shift perturbations (CSP). Residues E31, (S32 and D33, Y34 and S45, A46 and R47, and A48 and R71), E85, and L88 have relatively high levels of CSP (about 2-fold higher than the average shift levels). These residues form two oppositely charged clusters in the structure: a negatively charged region in the stem of the protein containing residues E31, D33, and E85 and a positively charged region at the head of the protein containing R47 and R71. Positively charged surface regions are colored blue, while negatively charged regions are colored red.

**FIG 3:**
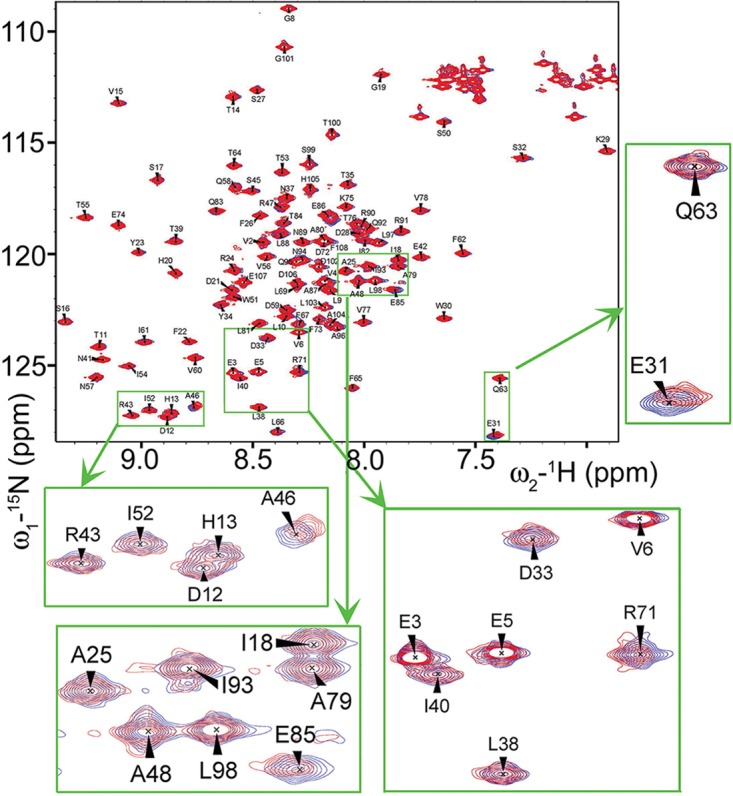
The presence of CsgA has little effect on the backbone amide residues of CsgE. The figure presents overlaid images of ^1^H-^15^N HSQC spectra of ^15^N-labeled CsgE W48A/F79A (80 µM) incubated in the presence (red) and absence (blue) of unlabeled CsgA (80 µM) for 24 h. The green boxes illustrate a few residues (such as E31, D33, R71, A46, and E85) with relatively higher chemical shift perturbations than others upon the addition of CsgA.

To identify residues responsible for mediating the CsgE-CsgA interaction (Fig. 1A), we examined the NMR chemical shift perturbation of all assigned CsgE W48A/F79A peaks in the presence and absence of CsgA. We identified 11 residues (E31, S32, D33, Y34, S45, A46, R47, W48, R71, E85, and L88) that exhibited a greater-than-2-fold-higher chemical shift perturbation than average, suggesting that these residues are involved in the CsgE-CsgA interaction (Fig. 2A to C). The four residues with the greatest chemical shifts cluster into two solvent-exposed and oppositely charged patches on CsgE (Fig. 2D). Residues R47 and R71 are located in the β_1_-β_2_ and β_3_-α_2_ loops at the “head” of CsgE, coalescing to form a patch of positive charge (33). Conversely, residues E31 and E85 contribute to a negatively charged patch at the “stem” of CsgE near the intrinsically disordered tails. A global comparison of the spectra in the presence and absence of CsgA demonstrates a high degree of overlap, suggesting that the backbone amides of ^15^N-labeled CsgE W48A/F79A are not perturbed upon addition of CsgA (Fig. 3).

To verify that the observed chemical shifts were not an artifact unique to the CsgE W48A/F79A protein, we also conducted chemical shift perturbation studies with WT CsgE in the presence and absence of CsgA (see Fig. S1 in the supplemental material). The two-dimensional (2D) ^1^H-^15^N HSQC spectrum of WT CsgE overlapped that of CsgE W48A/F79A, allowing partial chemical shift assignment (33). As with the CsgE W48A/F79A spectrum, small perturbations in the chemical shifts were detected upon addition of CsgA that corresponded to residues E31, R71, and R47, indicating that these interactions are not unique to the monomeric double mutant.

10.1128/mBio.01349-18.1 FIG S1  Overlay of ^1^H-^15^N HSQC spectra of ^15^N-labeled WT CsgE (80 µM/0.4 M arginine) in the presence (red) and absence (blue) of unlabeled CsgA (80 µM). Residues E31, A46, R47, and R71 were assigned based on the chemical shift of the double mutant CsgE W48A/F79A. Download FIG S1, TIF file, 0.8 MB. Copyright © 2018 Klein et al. 2018 Klein et al. This content is distributed under the terms of the Creative Commons Attribution 4.0 International license.

### Functional validation of charged residues by site-directed mutagenesis.

**FIG 4:**
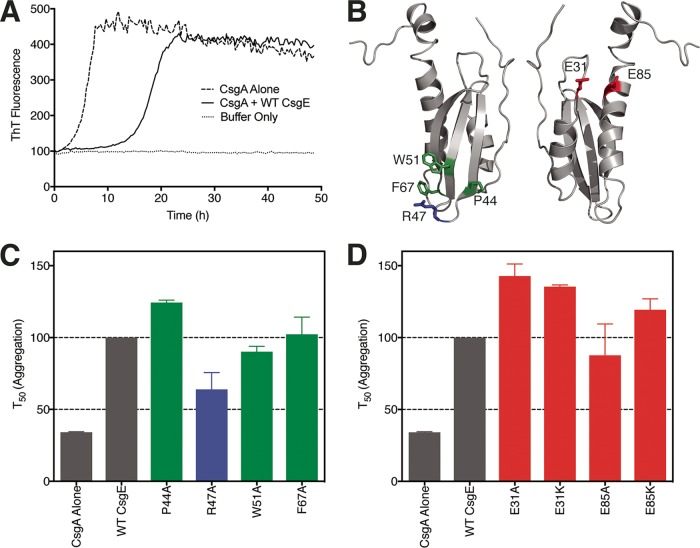
Mutation of charged residues differentially affects the CsgE-CsgA interaction. (A) CsgA (20 μM) was incubated in the presence (solid line) and absence (dashed line) of CsgE at 25°C. ThT fluorescence was measured every 20 min for 48 h to determine the extent of CsgA fiber polymerization. Consistent with previous reports, the presence of CsgE disrupts the aggregation of CsgA into fibers. (B) Mutations were made to positively charged and nonpolar residues at the head of CsgE and to the negatively charged E31 and E85 residues in the stem. Positively charged residues are colored blue, negatively charged residues are colored red, and uncharged residues are colored green. (C) Time necessary for CsgA to reach 50% of maximal aggregation (*T*_50_) when incubated with each purified mutant, as measured by Thioflavin T fluorescence. The *T*_50_ of CsgA autopolymerization was decreased by over 50% in the CsgE^R47A^ variant relative to WT CsgE, while mutation of nearby uncharged residues had a much more subdued effect. (D) Introduction of positively charged residues near the tail of the CsgA molecule increased the *T*_50_ of CsgA aggregation.

CsgE can prevent purified CsgA from aggregating into an amyloid fiber (30). To investigate the role of the positively charged head and negatively charged “stem” patches in the CsgE-CsgA interaction, we examined the effect of mutations in the head and stem residues of CsgE on polymerization of purified CsgA *in vitro* using the Thioflavin T (ThT) assay (8, 33). Addition of recombinant WT CsgE to freshly purified CsgA at a substoichiometric ratio of 1:4 CsgE/CsgA increased the time necessary for CsgA to reach 50% of its maximal aggregation (*T*_50_) (Fig. 4A). CsgE R47A was incubated with CsgA at 25°C for 48 h, and ThT fluorescence was measured in 20-min intervals. No changes in the overall thermal stability of CsgE were observed for any of the mutants (Fig. S2). We anticipated that disruption of the CsgE-CsgA interaction would shorten the observed *T*_50_, and indeed, CsgE R47A was less efficient than WT CsgE in extending the lag phase of CsgA (Fig. 4C). To determine if this effect was charge and residue specific, nearby nonpolar residues were also mutated to alanine, and the resultant protein was assessed for its ability to delay CsgA aggregation. CsgE W51A and CsgE F67A performed similarly to WT CsgE, and CsgE P44A prolonged the lag phase relative to WT CsgE (Fig. 4C). The E31 and E85 residues in the stem of CsgE were each mutated to both an alanine and a lysine to similarly investigate the role of the stem in mediating the CsgE-CsgA interaction. CsgE E85K, E31A, and E31K all demonstrated an increased ability to extend the lag phase of CsgA (Fig. 4D). Thus, the R47 head residue makes a critical charge-charge interaction with CsgA that is important for its inhibition of CsgA amyloidogenesis, while the E31 and E85 residues temper the CsgE-CsgA interaction by making the surface change of the CsgE molecule more negative.

10.1128/mBio.01349-18.2 FIG S2  Thermal stability of select CsgE mutants. Thermal stability was assessed via differential scanning fluorimetry using Sypro orange hydrophobic dye. *T*_*m*_ was determined by identifying the inflection point from the sigmoidal region of the melting curve. Download FIG S2, PDF file, 0.03 MB. Copyright © 2018 Klein et al. 2018 Klein et al. This content is distributed under the terms of the Creative Commons Attribution 4.0 International license.

### The CsgE-CsgA interaction is necessary, but not sufficient, for *in vivo* curli fiber formation.

**FIG 5:**
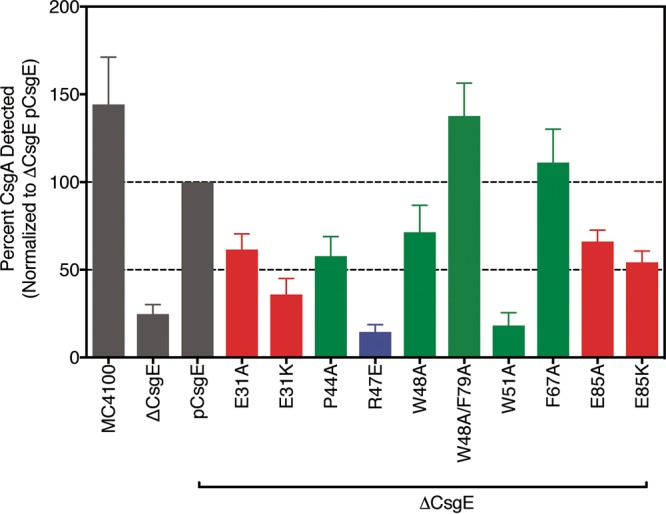
Quantification of curli fiber formation on the bacterial surface. Curli fiber formation was measured by Western blotting densitometry following treatment of cells with hexafluoroisopropanol (HFIP) to depolymerize curli fibrils.

Because CsgE is required for curli biogenesis *in vivo* (30), we tested the ability of the CsgE head and stem mutants to mediate curli formation by ectopically expressing *csgE* mutants in E. coli MC4100 *ΔcsgE*. Curli expression was quantified from cells grown on solid yeast extract-Casamino Acids (YESCA) agar at 30°C for 40 h. Colonies were isolated, treated with hexafluoroisopropanol (HFIP) to depolymerize curli fibrils, and subject to Western blot analysis to measure total CsgA levels (34). The total levels of CsgA detected in MC4100 *ΔcsgE* mutants were decreased 4-fold relative to those detected in WT MC4100 cells, demonstrating that CsgA was not accumulated at high levels in the periplasm (Fig. 5). Mutation of positively charged head residue R47 (CsgE R47E) or hydrophobic residue W51 (CsgE W51A) reduced detectable CsgA levels to those seen with a *csgE* null mutation, indicating a complete loss of CsgE function during curli biogenesis (Fig. 5). CsgE P44A also showed reduced CsgA levels relative to WT CsgE but not to the same degree as a *ΔcsgE* mutant. Similarly, the ectopic expression of each of the CsgE E31A, CsgE E85A, CsgE E31K, and CsgE E85K mutants facilitated less curli production than WT CsgE (Fig. 5). The monodisperse double mutant, CsgE W48A/F79A, was able to fully complement curli biogenesis in a *ΔcsgE* background (Fig. 5).

### The CsgE-CsgG interaction is impervious to disruption by a single point mutation.

**FIG 6:**
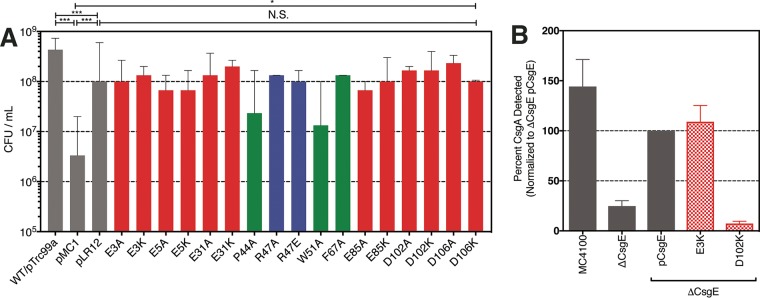
Mutation of negatively charged residues in the intrinsically disordered “tails” of CsgE does not abrogate the CsgE-CsgG interaction. (A) MC4100 *Δcsg* cells overexpressing CsgG from pMC1 were complemented with pTRC99a encoding WT and mutant CsgE. Each variant tested, including negatively charged residues in the N- and C-terminal IDRs, were able to rescue cells from CsgG-mediated toxicity. *, *P* < 0.05; ***, *P* < 0.001; N.S., not significant. (B) CsgE D102K, but not CsgE E3K, blocks curli assembly on the outer membrane of the cell.

Overexpression of CsgG in E. coli has been shown to lead to toxicity in the presence of bile salts (23). Coexpression of CsgE rescues this toxicity, suggesting that CsgE is able to gate the CsgG channel (20, 23). Thus, we used a bile salt sensitivity assay to probe the CsgE-CsgG interaction, hypothesizing that CsgE mutants with a disrupted CsgE-CsgG interaction would be unable to rescue E. coli overexpressing CsgG from bile salt-induced toxicity. The negatively charged tail residues in CsgE (E3, E5, D102, and D106) were mutated to alanine or lysine, and the mutants were expressed in E. coli MC4100 *Δcsg* cells along with *csgG* in the presence of bile salts. In addition, the mutations in the head and stem which affected the CsgE-CsgA interaction were also tested in this assay. All of the mutants were stably expressed (Fig. S3). Serial dilutions of cells harboring these mutations were plated on McConkey agar supplemented with 2% (wt/vol) bile salts, and growth was quantified after 48 h of incubation at 26°C (Fig. 6A). We found that all of the mutants tested were able to rescue cells from bile salt sensitivity, suggesting they did not interrupt the CsgE-CsgG interaction required for gating the channel (Fig. 6A). However, in the curli biogenesis assay described above, we found that a D102K mutation resulted in a nearly complete loss of curli assembly (Fig. 6B) whereas the E5K mutation had no effect.

10.1128/mBio.01349-18.3 FIG S3  Introduction of selected point mutations has minimal effects on CsgE stability *in vivo*. Cells harboring the pLR12 plasmid were induced with IPTG for 30 min. Cells (100 μl) normalized to an OD_600_ of 1.0 were pelleted and resuspended in 2× SDS sample buffer. Anti-hemagglutinin (anti-HA) and anti-CsgG antibodies were used to detect the CsgE and CsgG proteins, respectively. Download FIG S3, PDF file, 0.1 MB. Copyright © 2018 Klein et al. 2018 Klein et al. This content is distributed under the terms of the Creative Commons Attribution 4.0 International license.

### CsgE oligomerization is time and temperature dependent.

**FIG 7:**
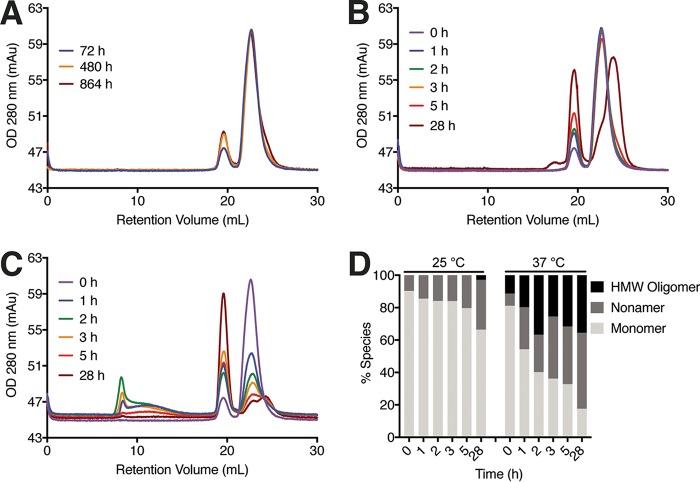
Temperature- and time-dependent oligomerization of CsgE monitored by size exclusion chromatography (SEC). (A to C) Representative results of purified CsgE (20 µM) incubated as a function of time are shown for three temperatures: 4°C (A), 25°C (B), and 37°C (C). (D) Relative distribution of each species at 25°C and 37°C as a function of time. HMW, high molecular weight.

Previous reports have provided structural and biochemical evidence of CsgE’s propensity to adopt a variety of oligomeric forms (Fig. 7A) (31, 33). To understand the influence of key residues on the kinetics of this process, we characterized the effect of point mutations on the oligomerization states of CsgE. Consistent with previous reports, we observed the partitioning of WT CsgE into two distinct peaks on a Superdex 200 10/300 column immediately following initial purification (Fig. 7A) (33). The two primary species that were present corresponded to monomeric CsgE (>90% by peak area) and a small amount of nonamer. This distribution remained stable for 3 days, with a moderate shift toward the nonameric form occurring in the subsequent 17 days at 4°C.

To understand the variables affecting the dynamic process of CsgE assembly, we conducted additional size exclusion chromatography (SEC) experiments on WT CsgE at different incubation times and temperatures. When incubation was performed at 25°C, the shift to the nonameric species occurred over a much shorter time scale than at 4°C. After 5 h at 25°C, the nonamer peak area was approximately 20% of the monomer peak area, and after 28 h, the nonamer peak area was approximately 40% of the monomer peak area (Fig. 7B). At 37°C, the monomeric peak loses >65% of its area within 1 h of incubation. The oligomeric species formed during this time included both a nonameric species and other larger oligomers (Fig. 7C and D).

**FIG 8:**
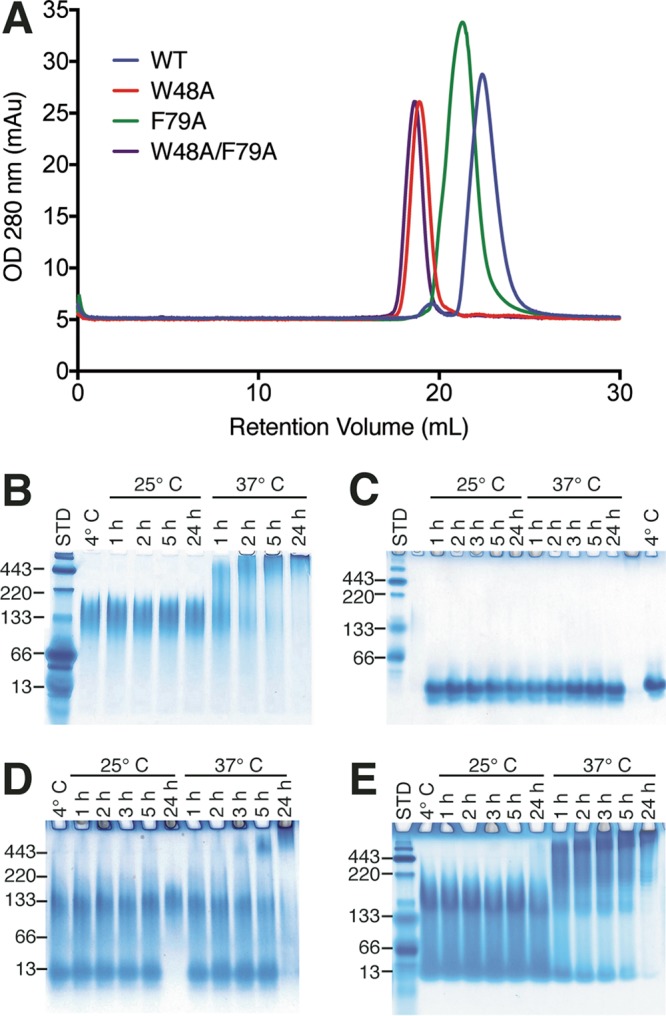
Mutations to aromatic head residues alter CsgE oligomerization kinetics. (A) Despite the observed shifts on the native-PAGE gel, both the single and double mutants appear as monomers on a Superdex 200 10/300 column. (B) Native-PAGE analysis demonstrates that WT CsgE is primarily present as a nonamer at 4°C and 25°C, but begins to form higher-molecular-weight oligomers following incubation at 37°C. (C) CsgE W48A/W79A remains exclusively monomeric at all temperatures and incubation times tested. (D) CsgE F79A is present as both a monomer and nonamer at 25°C, with a pronounced shift to higher oligomers at 37°C. (E) W48A remains a mixture of monomers and nonamers during short incubation times at 25°C and 37°C, with the emergence of the higher-molecular-weight species only occurring after prolonged incubation at 37°C.

Tracing of WT CsgE by size exclusion chromatography coupled to multiangle light scattering (SEC-MALS) revealed four distinct species: two forms of monomer; a small oligomer; and high-molecular-weight oligomers and aggregates (Fig. S4A). Partitioning of the monomer peak into monomeric and nonameric species was observed when it was rerun over a size exclusion column (Fig. 7A). In contrast, CsgE W48A/F79A appeared to monodisperse on SEC and ran as a single monomeric band on a native-PAGE gel. The SEC-MALS tracing revealed a single dominant peak with a calculated molecular weight of 14.5 kDa (Fig. S4B). To parse the individual contributions of W48 and F79 to the temperature-dependent oligomerization of CsgE, the CsgE W48A and CsgE F79A mutants were analyzed via SEC and native-PAGE. Both CsgE W48A and CsgE F79A appeared monomeric by SEC, although they were differentially retained on the column relative to both WT CsgE and the molecular weight standards (33) (Fig. 8A). On native PAGE gels, purified WT CsgE appeared primarily nonameric at 4°C and 25°C with a shift to high-molecular-weight oligomers at 37°C (Fig. 8B). CsgE W48A/F79A was monomeric at all temperatures and time points tested (Fig. 8C). Following short incubation times at 4°C and 25°C, CsgE W48A segregated into two bands corresponding to the monomer and nonamer, with the monomeric species disappearing after 24 h at 25°C (Fig. 8D). At 37°C, higher-molecular-weight species began to appear after 5 h and dominated the distribution (Fig. 8D). In contrast, CsgE F79A was comparatively more heterogeneous at 4°C and 25°C and demonstrated a pronounced shift to high-molecular-weight aggregates after only an hour of incubation at 37°C (Fig. 8E). Thus, both W48 and F79 contributed to the oligomerization of CsgE at 4°C and 25°C but W48 drove the formation of the high-molecular-weight CsgE species at 37°C.

10.1128/mBio.01349-18.4 FIG S4  CsgE W48A/F79A exists as a monodisperse monomer. Elution profiles of WT CsgE (solid black) and CsgE W48A/F79A (solid blue) were determined by size exclusion chromatography coupled to multiangle light scattering (SEC-MALS) using a Superdex 200 Increase 10/300 GL column. Calculated molecular masses from each peak of each trace are shown as dotted lines. WT CsgE elutes in four peaks: a large, high-molecular-weight aggregate representing 32% of the loaded mass; a small intermediate species corresponding to a small oligomer (2% of the mass); and two lower-molecular-weight species with calculated masses of 13.2 and 19.6 kDa, respectively. In contrast, CsgE W48A/F79A is present almost exclusively as a monomer, with a calculated molecular weight of 14.5 kDa. Download FIG S4, PDF file, 0.05 MB. Copyright © 2018 Klein et al. 2018 Klein et al. This content is distributed under the terms of the Creative Commons Attribution 4.0 International license.

### Key residues for CsgE function are subject to selection within the E. coli species.

**FIG 9:**
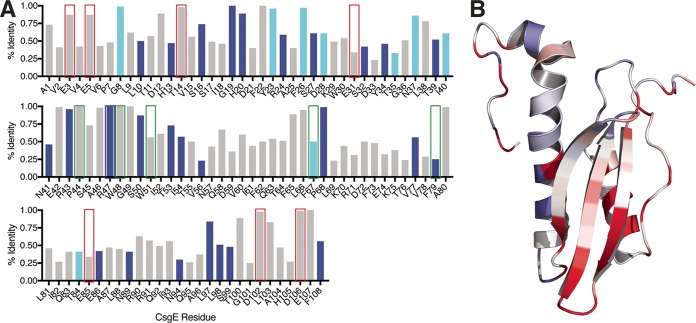
Residues throughout the CsgE molecule are subject to variable selection pressures. (A) Residues subject to negative selection occur preferentially on the core and at the head of the CsgE protein. Bars shown in dark blue correspond to strong purifying selection (*P* < 0.05), while bars shown in light blue correspond to 0.10 > *P* > 0.05. (B) Residues that are more conserved across Gram-negative bacterial species are shown in red, while residues that are less conserved are shown in blue.

To better understand the evolution of the *csgE* gene throughout all bacterial phyla, we examined the sequences of CsgE homologues in the EMBL-EBI database with an E value of <10^−10^. A total of 397 CsgE amino acid sequences representing 3,388 nucleotide sequences were aligned using the multiple-sequence alignment based on fast Fourier transform (MAFFT) FFT-NS-I ×2 algorithm (35). Overall pairwise identity for this sequence set was 58.7%, with an overall pairwise similarity of 72.2%. Pairwise identity by residue is indicated by the height of the bars in Fig. 9A and is mapped onto the CsgE structure in Fig. 9B. These findings reveal a cluster of conservation near the head of CsgE, as well as select residues in the negatively charged, intrinsically disordered tails. Of note, R43 and R47 have pairwise identities of 96% and 100%, respectively, while each of residues E3, E5, D102, D106, and D107 within the intrinsically disordered tails has a pairwise identity of >85% (Fig. 9A). This supports our data indicating that the concentration of the positive charge at the head of CsgE and the concentration of the negative charge in the tail regions of CsgE contribute to CsgE function. We also find that each of the E31 and E85 stem residues has a pairwise identity of <35%, suggesting that the CsgE protein is more tolerant of variation at these residues.

To examine the selective pressures acting upon the *csgE* gene within the E. coli species, we obtained the nucleotide sequences for 1,985 *csgE* homologues across all E. coli genomes in the EMBL-EBI Bacterial Genomes database. A total of 41 unique amino acid sequences remained following removal of identical sequences. The mature amino acid sequences demonstrated an overall pairwise identity of 91.3% and an overall pairwise similarity of 93.3%. The ratio of synonymous to nonsynonymous mutations (dS/dN) was examined to better identify the selection pressures acting on each residue within the *csgE* gene. Negative/purifying selection was found at 27 sites with a Bonferroni-corrected *P* value of less than 0.05 (indicated by dark blue in Fig. 9A). Nine additional sites were identified with a Bonferroni-corrected *P* value of between 0.05 and 0.1 (indicated by light blue in Fig. 9A). No sites of positive selection were identified. While the majority of the negatively selected residues faced inward to contribute to the core fold of CsgE, R47 and R43 were surface exposed and subjected to strong purifying selection (Fig. 9B). Conversely, E31 and E85 were not subjected to selective pressure, again indicating that some variability in these residues is tolerated.

## DISCUSSION

CsgE is a periplasmic curli assembly protein required for the transport of amyloidogenic CsgA to the CsgG pore (31). The NMR structure of a stable CsgE W48A/F79A mutant reveals a three-stranded anti-parallel β-sheet core flanked by three α-helices (33). In this study, we used NMR chemical shift perturbation to identify residues that participate in the CsgE-CsgA and CsgE-CsgG interactions. The roles of these residues were validated using *in vitro* and *in vivo* assays of CsgE function. We identified a region of positive charge formed by the confluence of the β_1_-β_2_ and β_3_-α_2_ loops at the head of CsgE that plays a key role in the CsgE-CsgA interaction and is required for curli assembly (Fig. 4C and 5). The identity of residue E31 in the α_1_-β_1_ loop and of residue E85 in α_3_ in the stem of CsgE modulates the ability of CsgE to prevent CsgA aggregation *in vitro* and to assemble curli fibers *in vivo* (Fig. 4D and 5). Residue D102 in the negatively charged C-terminal tail of CsgE contributes to curli assembly without disrupting the CsgE-CsgG interaction (Fig. 6).

The finding that charge-charge interactions underlie interactions within amyloidogenic proteins is consistent with existing literature (20, 36). During curli biogenesis, the periplasmic CsgC chaperone is known to prevent inappropriate periplasmic CsgA polymerization through a series of electrostatic interactions, instead targeting CsgA for degradation in the event of disrupted subunit secretion (22). Computational and *in vitro* analyses of charged amino acids in amyloid beta have revealed the importance of electrostatic interactions in oligomerization outside the curli system (36). We demonstrated that the CsgE R47A head mutant showed a marked decrease in its ability to protect CsgA from aggregation and that disruption of the R47 residue abrogated the ability of CsgE to assemble curli fibers without affecting degradation of CsgA in the periplasm (Fig. 4A and 5). Coupled with the invariance of this residue across all Gram-negative bacterial species, these data suggest that a specific charge-charge interaction occurs between the head of CsgE and CsgA. This finding supports the “head-centric” model of complex formation in which the positively charged head groups of CsgE coalesce in the center of the nonameric cap to bind CsgA and facilitate its transport to the CsgG pore.

NMR perturbation of the CsgE chemical shifts in the presence of CsgA also revealed a novel charge-charge interaction between the stem of CsgE and CsgA (Fig. 2). Introduction of the CsgE E31K and CsgE E85K mutations resulted in a 60% reduction in curli fiber assembly despite an augmented ability to delay the lag phase of CsgA polymerization (Fig. 4D and 5). These findings suggest that the surface charge in the stem of CsgE controls the balance between CsgA association and dissociation, and the low degree of conservation at residues 31 and 85 suggests that CsgE can tolerate substitutions at those positions.

CsgE harboring mutations in the head, stem, and tail regions retained the ability to block passage of small molecules through the CsgG pore (Fig. 6). The persistence of this interaction despite disruption of charged residues is consistent with a previous report that mutations disrupting the formation of the nonameric CsgE cap *in vitro* were still able to complement curli biogenesis *in vivo* (33). In this study, we demonstrated that CsgE W48A/F79A is unable to form nonameric and high-molecular-weight species in a temperature-dependent manner *in vitro*. We also demonstrated that the W48A and F79A mutations individually shift the CsgE population to the monomeric species. The persistence of *in vivo* curli assembly despite an observed attenuation of *in vitro* oligomerization with CsgE W48A/F79A suggests that curli biogenesis may be affected by the presence of other curli proteins or periplasmic factors that are not present *in vitro*.

In sum, these data demonstrate that positively charged residues in the “head” of CsgE mediate specific charge-charge interactions with CsgA, supporting the head-centric cryo-EM fitting model described previously (30). We also found that negatively charged stem residues modulated CsgE binding to CsgA, possibly to maintain a finely tuned equilibrium that promotes CsgA transport and secretion. This balance of specific and nonspecific charge-charge interactions presents a stark contrast to known protein cages such as the GroEL/GroES chaperonin and ABC toxin complexes. In these systems, the oligomeric cages recognize hydrophobic residues within a broad array of client proteins prior to encapsulation to facilitate their sequestration and promote protein folding (37–39). The insights into the structural basis of CsgE function enhance our mechanistic understanding of bacterial amyloid assembly during biofilm formation and provide insight into a mechanism of protein recognition distinct from those currently known to occur in the bacterial periplasm.

## MATERIALS AND METHODS

### Strains and plasmids.

6×-His-tagged CsgE expression constructs containing single amino acid substitutions were generated in plasmid pNH27 (MC4100 CsgE in pTRC99a) using the QuikChange protocol (30, 40) and were transformed into E. coli C600. For *in vivo* curli biogenesis studies, WT CsgE from E. coli MC4100 was cloned into pTRC99a encoding the pBR322 origin of replication. Mutations were generated using a QuikChange system. For bile salt sensitivity studies, mutations in CsgE were made in pTRC99a encoding CsgE and CsgG (pLR12) using the QuikChange protocol. Plasmids harboring mutations were transformed into MC4100 *ΔcsgE* (LSR11) cells (24). 6×-His-tagged CsgA lacking the 20-residue N-terminal signal sequence was expressed in the cytoplasm of NEBC2566 cells containing the pET11d-derived pNH11 plasmid (34).

### NMR spectroscopy.

^15^N-labeled CsgE for use in NMR spectroscopy was generated as previously described using M9 minimal medium containing 1 g/liter of [^15^N]ammonium sulfate (33). Deuterium oxide (D_2_O) and [^15^N]ammonium sulfate were obtained from Cambridge Isotope Laboratories. CsgE (WT or W48A/F79A) (80 μM) was incubated in the presence or absence of 80 µM unlabeled CsgA for 24 h. NMR spectra were collected on a Bruker Avance III 600-MHz spectrometer equipped with a cryogenic triple-resonance probe. The proton chemical shifts were internally referenced to 2,2-dimethyl-2-silapentane-5-sulfonic acid (DSS), and the chemical shifts of ^15^N were referenced indirectly to DSS using the absolute frequency ratios. All NMR data were processed using Bruker TopSpin 3.2. The chemical shift perturbations (CSP) were analyzed using the program NMRFAM-SPARKY (32). The average Euclidean distance change was calculated as $\sqrt{\frac{1}{2}}\left[\delta_{H}^{2}+\left(\alpha \cdot \delta_{N}^{2}\right)\right]$, where δ_*H*_ and δ_*N*_ represent the absolute chemical shifts of ^1^H and ^15^N, respectively, and the scale factor (α) is 0.14.

### Thioflavin T assays.

ThT CsgA polymerization assays were conducted as previously reported (8, 34). WT CsgE and mutant CsgE were purified from the lysate of E. coli C600 cells containing pNH27 by cobalt affinity chromatography (resin, Goldbio Product catalog no. H310). Following dialysis into 20 mM Tris (pH 8.0), samples were run over an anion exchange column (Mono Q, GE Healthcare) and eluted with an NaCl gradient. Samples were dialyzed into 50 mM KHPO_4_ (pH 7.3) and stored at 4°C. CsgA was purified from NEBC2566/pNH11 cells lysed in 50 mM KPi (pH 7.3) supplemented with 8 M guanidine hydrochloride for 24 h. Cellular debris was cleared by centrifugation, and the resulting supernatant was loaded onto a nickel-nitrilotriacetic acid (Ni-NTA) column. CsgA was eluted in 50 mM K_2_HPO_4_ (pH 7.3)–125 mM imidazole and filtered through a 4-ml Ultracel 30-kDa-molecular-weight-cutoff (MWCO) Amicon Ultra spin column (Millipore catalog no. YFC803024) to remove aggregates and oligomers. Monomeric CsgA was buffer exchanged into 50 mM K_2_HPO_4_ (pH 7.3) using a 5-ml, 7,000-MWCO Zeba spin desalting column (Thermo Scientific catalog no. 89892). The CsgA concentration was determined using the *A*_280_ value with a molar extinction coefficient of 11,460 M^−1^ cm^−1^.

Purified CsgA protein was immediately added to the wells of 96-well black polystyrene nonbinding plates (Corning catalog no. 3650) to a final concentration of 20 µM. CsgE was added to a final concentration of 5 µM, unless otherwise noted. Plates were incubated in a Tecan Infinite 200Pro plate reader for 48 h at 25°C. Reads were taken following 3 s of linear shaking every 20 min. The excitation and emission wavelengths were set to 438 nm and 495 nm, respectively. Values represent the averages and standard deviations of results from three technical replicates.

### Quantification of CsgA in curli-expressing cells.

MC4100 *ΔcsgE* cells complemented with WT or mutant *csgE* on a pTRC99a plasmid were grown overnight in LB supplemented with 100 µg/ml ampicillin. Each sample (5 μl) was spotted into a YESCA agar plate, which was incubated at 30°C for 40 h. Cells were scraped and resuspended in 1 ml of phosphate-buffered saline (PBS). Cell density was normalized using the optical density at 600 (OD_600_). Cells (200 μl) at an OD_600_ of 1 were pelleted. Cells were then resuspended in 100 µl hexafluoro-2-pronanol (HFIP) to depolymerize preformed curli fibers. Samples were then dried in a rotary evaporator and resuspended in 100 µl SDS sample buffer (60 mM Tris [pH 6.8], 5% β-mercaptoethanol, 10% glycerol, 3% SDS). Samples were run on a 15% SDS-PAGE gel, transferred to an Immobilon-P^SQ^ polyvinylidene difluoride (PVDF) membrane (Millipore catalog no. ISEQ00010), and developed using mouse anti-CsgA antibody. Quantification was performed using densitometry and Quantity One image analysis software. Significance was computed relative to MC4100 *ΔcsgE* complemented with WT pTRC99a-*csgE* on the appropriate control plasmid using the Mann-Whitney *U* test. All studies were conducted with a minimum of three independent replicates.

### Differential scanning fluorimetry.

The thermal stability of CsgE was assessed using differential scanning fluorimetry. Purified CsgE was dialyzed into 50 mM KHPO_4_ (pH 7.3). In addition to Sypro orange protein gel stain (Sigma catalog no. 5692), each well contained 2 µg of WT or mutant CsgE and buffer with and without 150 mM NaCl added to reach a final volume of 70 µl. Samples were heated from 20°C to 100°C in 30-s 0.5°C increments using a Bio-Rad C1000 thermocycler with a CFX96 reverse transcription-PCR (RT-PCR) attachment.

The melting temperature (*T*_*m*_) was determined by fitting the melt curves to Boltzmann's equation {*y* = *A*_2_ + (*A*_1_ − A_2_)/[1 + exp(*x* − *T*_*m*_)/*dx*]} (41). Reported melting temperatures represent results from an average of three technical replicates.

### Bile salt sensitivity assays.

MC4100 *Δcsg* cells were transformed with pTRC99a containing both WT *csgG* and WT or mutant *csgE* in the multiple-cloning site under the control of the *lac* operon (pLR12). Cells were grown to an OD of 0.1 in LB broth at 37°C with shaking and were pelleted by centrifugation at 6,500 rpm for 10 min. Cells were resuspended in 1× PBS to an OD of 1, and 3-µl volumes of each of eight serial dilutions were plated on McConkey agar plates supplemented with 2% bile salts, 100 µg/ml ampicillin, and 0.1 mM IPTG (isopropyl-β-d-thiogalactopyranoside). Plates were allowed to dry and then grown for 48 h at 26°C. Colonies were counted to determine OD.

### Native-PAGE and size exclusion chromatography.

CsgE used in SEC and native-PAGE analysis was expressed and purified as previously described (33). Briefly, WT CsgE and mutant CsgE were expressed from the pNH27 plasmid and subjected to an initial cobalt affinity column. The samples were run over a HiPrep 26/60 Sephacryl S-100 HR column (GE Healthcare) and eluted with 50 mM potassium phosphate–150 mM NaCl (pH 7.4). The peak corresponding to monomeric CsgE was isolated and used for subsequent SEC and native-PAGE experiments. SEC analysis of CsgE oligomerization was performed using a Superdex 200 10/300 column (GE Healthcare) and a flow rate of 0.5 ml/min. The elution buffer was 50 mM potassium phosphate–150 mM NaCl (pH 7.4). The results of SEC analysis performed at different temperatures were slightly shifted in profile and peak intensity. For the comparisons, we show only the results of SEC analysis carried out at room temperature (about 25°C) for CsgE preincubated at different temperatures (4, 25, and 37°C). Some degradation of CsgE was seen after 28 h of incubation at 25°C.

### SEC-MALS.

Oligomerization of CsgE was analyzed by size exclusion chromatography coupled with multiangle light scattering (SEC-MALS). WT CsgE and mutant CsgE were purified using two sequential cobalt affinity columns and dialyzed into 50 mM KHPO_4_ (pH 7.3) with and without 150 mM NaCl and concentrated to 2 mg/ml. Protein was injected into a Superdex 200 Increase 10/300 GL column (GE Healthcare catalog no. 28-9909-44) and run at a flow rate of 0.3 ml/min using an Agilent Technologies 1260 Infinity high-performance liquid chromatography (HPLC) system. Light scattering was measured using a Dawn Heleos II multiangle static light scattering detector (Wyatt Technology) coupled to an Optilab T-rEX refractometer (Wyatt Technology). Data were collected and analyzed using ASTRA software.

### Multisequence alignment and selective-pressure analysis.

To determine the evolutionary pressures acting on codons encoding individual amino acid residues within E. coli species and across all taxa of bacteria, a PHMMR (EMBL-EBI) search was conducted against the “Ensembl Genomes Bacteria” database using an E value cutoff of 1.0e−10. Nucleotide sequences were downloaded into two separate bins: one containing all CsgE homologues and one containing homologues found exclusively in E. coli.

For the E. coli-only search, 2,002/2,003 hits were downloaded from the EMBL-EBI database and loaded into Geneious R10 (42). Duplicate sequences were removed to generate a list of 93 unique E. coli sequences. Sequences with ORFs less than 300 nucleotides in length were then removed to generate a list of 80 sequences. These sequences were then translated into amino acid sequences and aligned using the MAFFT FFT-NS-I ×2 algorithm with the BLOSUM62 scoring matrix (35, 43, 44). These amino acid alignments were then translated into codon-based nucleotide alignments using the Pal2Nal webserver (45). Recombination analysis was performed using the Genetic Algorithm for Recombination Detection (GARD) method on the DataMonkey webserver (46–48). Positive/negative selection analysis was performed using the Fixed Events Likelihood (FEL) algorithm, a GARD-generated phylogenetic tree, and the HKY85 nucleotide evolution model (49).
