# Supplementary figures and images for: Structure-Function Analysis of the Curli Accessory Protein CsgE Defines Surfaces Essential for Coordinating Amyloid Fiber Formation
Source: mBio. 2018 Jul 17;9(4):e01349-18. doi: 10.1128/mBio.01349-18 (PMC6050966; doi:10.1128/mBio.01349-18)

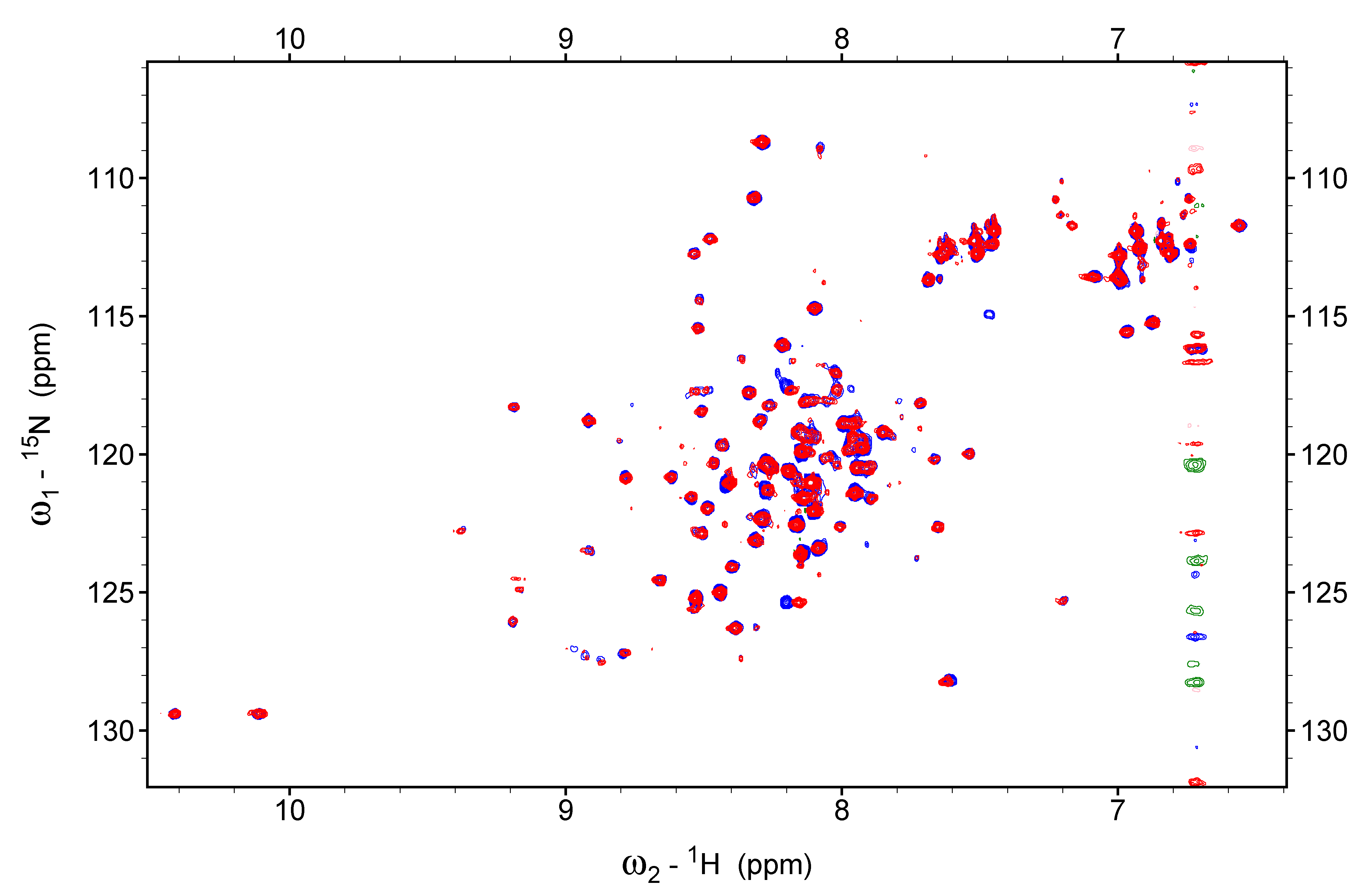

Supplement: FIG S1 [file mbo004183987sf1.tif]

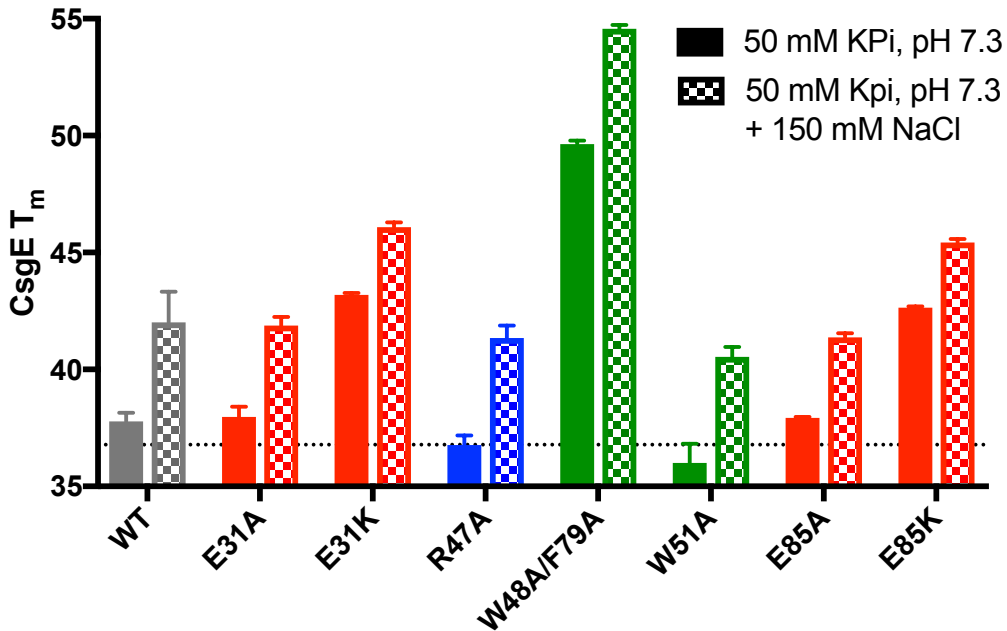

Supplement: FIG S2 [file mbo004183987sf2.pdf]

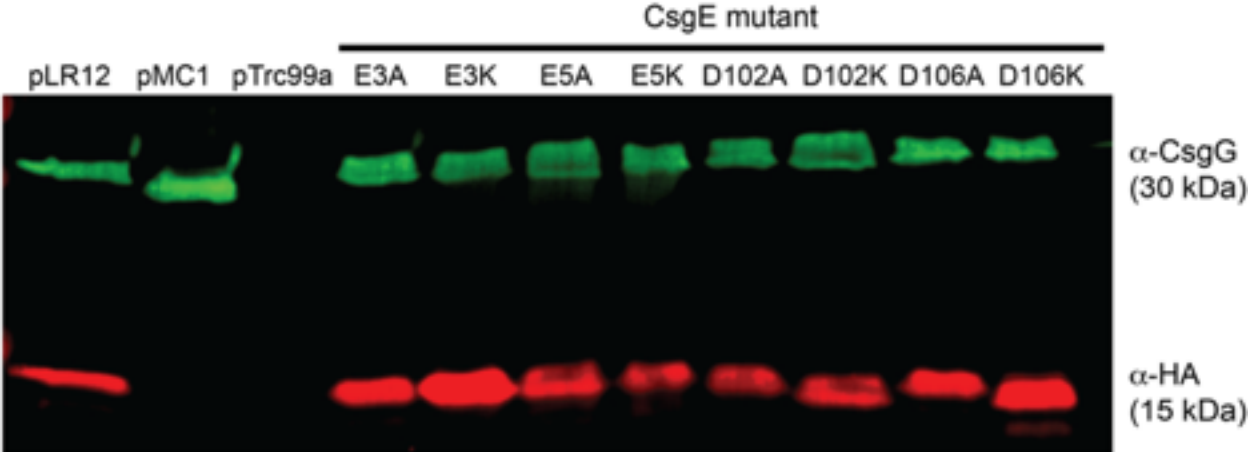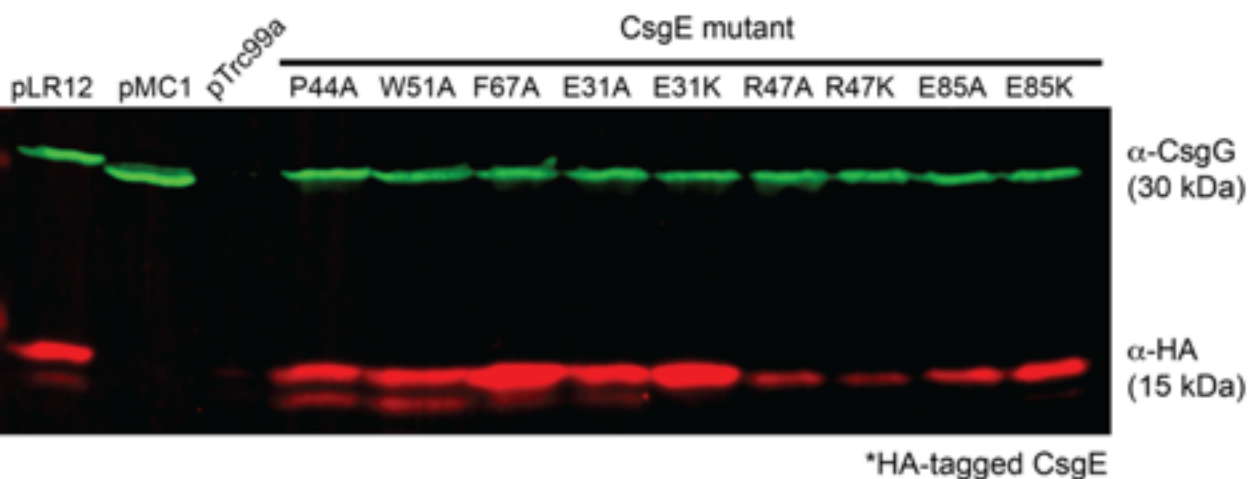

Supplement: FIG S3 [file mbo004183987sf3.pdf]

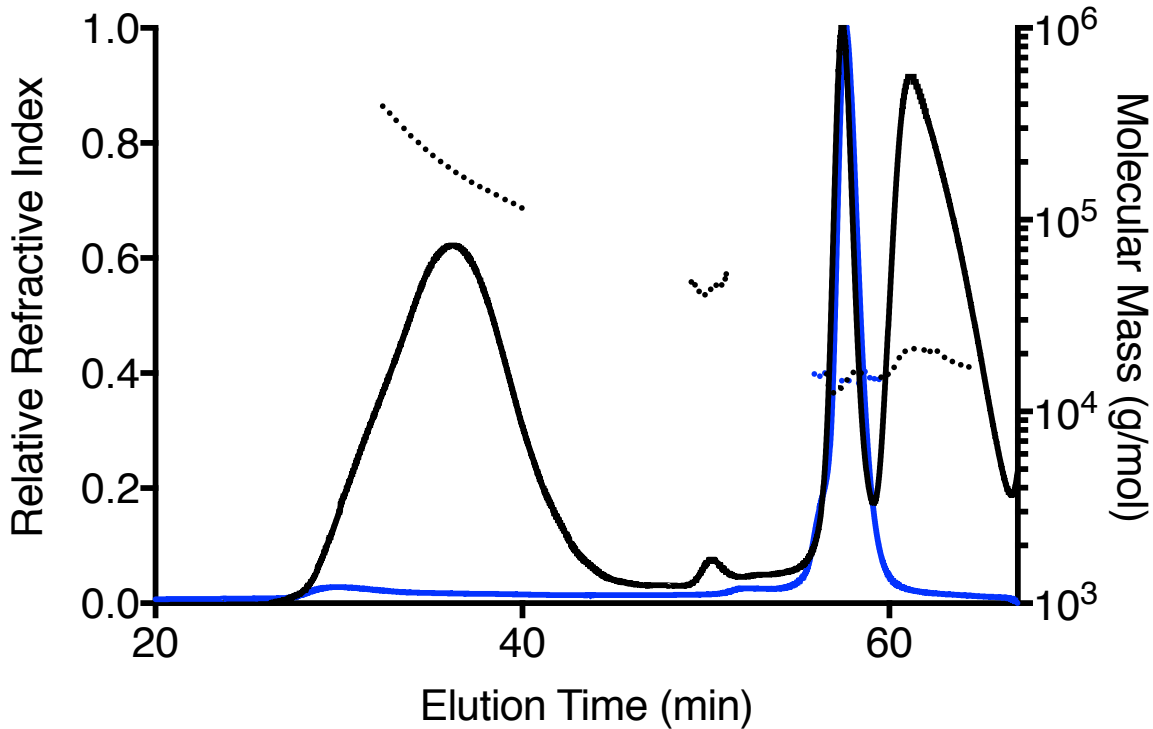

Supplement: FIG S4 [file mbo004183987sf4.pdf]
